# Remove or Reduce: Demotion, Content Moderation, and Human Rights

**DOI:** 10.1007/s10982-026-09557-8

**Published:** 2026-04-24

**Authors:** Jeffrey W. Howard, Beatriz Kira

**Affiliations:** 1 Political Science, University College London, 29-31 Tavistock Square, London, WC1H 9QU UK; 2 Law, University of Sussex, Freeman Building, Brighton, BN1 9QE UK

## Abstract

How should social media platforms respond to harmful speech? For some content, platforms respond by removing it. But for other content, platforms allow the speech to be posted, but reduce its distribution throughout their networks to reduce its visibility. This second technique—*demotion*—is swiftly becoming one of the most significant ways in which our communications are governed. Yet it strikingly remains normatively undertheorized. This article offers a framework for when platforms should demote content instead of removing it. After explaining why demotion policies by platforms stand in need of normative justification, and reviewing the various human rights norms that constrain how demotion should operate, it argues that demotion should be pursued instead of removal in cases where removal would violate principles of necessity or proportionality, yet demotion would satisfy them. By explaining when and why this is so, the article seeks to illuminate how notions of necessity and proportionality—central to both political philosophy and human rights law—should be applied to the distinctive context of online content moderation.

## Introduction

Social media platforms are among the most significant spaces of contemporary communication, constituting central forums in which people go to express themselves, engage with others, and receive information. Over time these platforms have developed elaborate rules regarding what speech is and is not allowed, with detailed policies setting out what kinds of content gets removed—from incitement to hate speech to bullying and beyond. Yet we no longer live in a world where ‘leave up or take down’ are the sole options available to platforms. Among the alternatives available to platforms is a technique often referred to as *demotion*. When content is demoted, users are allowed to post it, but its amplification is limited—reducing its distribution across the network and thus how many (and how often) people see it.^1^

For many years, platforms were coy about what content received this treatment—provoking an outburst of allegations of so-called ‘shadowbanning’. This public outcry had a salutary impact: platforms are now more transparent about the categories of speech that, while strictly permitted, receive demotion. Meta, for example, now has an official page listing ‘Types of Content We Demote’ on its Facebook and Instagram platforms—encompassing diverse categories ranging from ‘fact-checked misinformation’ (e.g. about climate change) to ‘unsafe reporting about suicide’.^2^ TikTok has a section outlining the ‘eligibility standards’ for its ‘For You’ recommender feed, pinpointing varieties of ‘problematic content’, such as ‘controversial weight management behaviors’, especially those that involve ‘promoting certain body types as ideal’.^3^ And X (formerly Twitter) has allegedly begun notifying individual users whose posts are labelled as ‘sensitive’, indicating that its ‘reach…may also be restricted, such as being excluded from the For You and Following timelines, recommended notifications, trends, and search results’.^4^

Despite this burgeoning practice, it remains deeply unclear how platforms should decide what, exactly, they should demote. If content is sufficiently objectionable that it should be reduced within the network, why not simply remove it? Likewise, if content is sufficiently innocuous that removal is uncalled for, why bother demoting it? While other scholars have offered astute analysis on how demotion and related processes operate,^5^ this topic has escaped the attention of political and legal philosophers.^6^ Our focus here is thus the *normative* one of when platforms ought to engage in demotion, and why.

In so doing, we invoke familiar normative principles about how interferences with people’s rights can be justified. For example, in some cases, removing content will be objectionable because it is *unnecessary,* as the relevant harm can be adequately addressed through the less invasive measure of demotion. This is so, we will explain, for content that becomes unacceptably harmful when encountered by audiences in high doses. Various forms of misinformation such as climate change denialism, as well as content promoting unhealthy body images, will fall under this analysis. In other cases, demotion is preferable to removal because removal is a disproportionately severe sanction (in a particular strict sense to be explained); for much mildly harmful content, the downside of removal outweighs the benefit. This explains the plausibility of demoting so-called ‘borderline content’—content which comes close to violating a content prohibition but falls short of doing so.

In Part 2, we review what demotion is, contrasting it with closely related phenomena. After reviewing why demotion policies stand in need of normative justification, Part 3 reviews the relevance of core human rights principles bearing on the governance of online speech. Part 4 discusses principles of legitimacy, legality, and suitability. Part 5 discusses necessity, and Part 6 turns to proportionality *stricto sensu*.

## Demotion, Algorithmic Deamplification, and Shadowbanning

*Demotion* refers to platform policies whereby designated categories of content are made less visible to users—such as by reducing or even excluding such content in recommendations, or by lowering such content’s place in rankings.^7^ Where a platform demotes some category of content, its visibility is deliberately reduced on such surfaces as Facebook’s Newsfeed, YouTube’s homepage and ‘Up Next’ feature, and TikTok’s ‘For You’ page. Such a practice has the aim of reducing the visibility of content, reducing how many—and how often—people see it, while still allowing it to be posted and discovered. 8

Demotion should be distinguished from a closely related phenomenon, which we will label *algorithmic deamplification—*the kind of visibility reduction that occurs within ordinary processes by which platforms curate content. While these terms are often used interchangeably, and their underlying technical processes can be similar, we argue this is misleading. Social-media platforms continually amplify and deamplify content as a matter of regular course, based on algorithmic predictions of what users will find more or less engaging. Yet such a practice of algorithmic deamplification, as part of the overall *curation of content*, differs from demotion as a form of *content moderation* in several respects.

One difference is that, when algorithmic deamplification occurs, it is generally *user-tailored*; the same content amplified to one user (e.g., sports content or stand-up humor) will be made less visible for another user. In contrast, demotion is generally *categorical*; for example, when a platform demotes climate misinformation, it is demoted *for everyone*.

But the crucial difference is that, when content is algorithmically deamplified based on a prediction of likely engagement, there is no accompanying substantive judgment about the merits of the content itself. Consider the point outside the social media context. When Netflix’s recommender algorithm declines to suggest a particular movie to you on the grounds that it predicts you won’t enjoy it, Netflix is not expressing the view that the movie is *bad* (indeed it may simultaneously be recommending the same movie to many others). Rather, Netflix is simply making a prediction that it won’t be to your taste. Ditto when social media platforms algorithmically deamplify content. In this way, algorithmic decisions are *viewpoint-neutral*. In contrast, demotion policies are justified on the basis of a substantive judgment that the demoted content is harmful or otherwise undesirable. Demotion policies, in other words, are often *viewpoint-discriminatory*—a point with normative significance, as we shall see in the next section.

Algorithmic amplification systems and demotion policies are certainly related. After all, demotion is relative to a baseline, what we can call ‘ordinary treatment’, whereby content is amplified (more or less) based on the ordinary features of the engagement-optimizing algorithmic system.^9^ In this way, demotion cannot be understood or even defined without reference to the broader system in which content visibility is algorithmically curated. They are normatively related, too: it is precisely because users are likely to find certain ‘problematic’ content engaging, such that the system will amplify it to them when left to its own devices, that demotion of that content may become appropriate. This, in fact, was Mark Zuckerberg’s own rationale for demoting so-called borderline content on Facebook and Instagram (content that comes close to violating content rules).^10^ Thus, demotion—like all content moderation policies—constrains the ordinary operation of the engagement-optimization algorithmic system.

Still, they remain district systems with important differences. Some differences are technical; whereas algorithmic curation involves the ordering of content determined by ‘engagement-based relevance scores’, demotion involves ‘ex post adjustments’ to those scores for content moderation purposes.^11^ Another illustrative difference is operational: demotion is understood as part of the process of content moderation handled by ‘trust-and-safety’ or ‘integrity’ teams, whereas curation is a concern engaging the product design and core business operations of the companies. The difference that matters most for our purposes here is that these systems are distinct *normatively*. Demotion policy, like removals policy, is properly evaluated by asking what duties platforms have to prevent harm, consistent with duties to support free expression. As Gillespie has argued, demotion belongs to the domain of content moderation, understood as enforceable rules on users’ behavior; while removing content and suspending users are the most severe sanctions, they are not the only sanction.^12^ In contrast, questions of algorithmic curation involve distinct normative questions about what values beyond engagement platforms should be designed to optimise.^13^

Having distinguished demotion from algorithmic deamplification, one final preliminary point is apt. While we shall use ‘demotion’ as an umbrella term for deliberate visibility reduction of certain categories of content, there are various techniques of demotion that might be deployed. For example, content can be *downranked* so that the system is less likely to recommend it to you. Content can be *excluded* from recommendations entirely. Or it can be *delisted* so that the content will not show up in a search; or *downlisted* within search results. Or it can be restricted in some other way—for example, so that only one’s followers will see one’s content.^14^ What exact technique (or indeed degree) of demotion is suitable depends largely upon the goal of demotion in any given platform policy area—a point to which we will return. In the meantime, we will use the term ‘demotion’ to encompass all such content moderation techniques of visibility reduction.

## Justifying Demotion

### Why Justification is Required

Do demotion policies stand in need of normative justification? We might think that the answer is ‘no’. As the popular slogan goes: ‘free speech is not free reach’.^15^ When a platform demotes content, it declines to amplify it – what is so bad about that? As we have already discussed, platforms *automatically* decline to amplify content all the time anyway, just on the basis of user-tailored engagement instead of any categorical policy. If *that* raises no normative difficulties for our freedom of expression, why does this?

To grasp the free speech issue most starkly, start by imagining that *the state* were to compel a platform to demote certain content. Suppose it were to compel platforms to demote all content promoting the Muslim faith, or promoting Catholicism. Or imagine the state were to compel platforms to demote all expression of pro-Republican viewpoints in the run-up to American national elections. Or imagine it compelled platforms to demote all videos defending LGBTQIA+ rights. Such compelled demotion would be incompatible with freedom of expression; it would be no adequate defense for the state to reply: ‘But we’re not *removing* the content; we’re only *demoting* it’. The fact that such hypothetical policies would involve a less severe sanction than removal does not obviate the free speech issue.^16^ The reason is that such policies would be *viewpoint-discriminatory*: they would suppress certain disfavoured viewpoints in order to limit people’s exposure to them. Yet the suppression of viewpoints is paradigmatically *prima facie* incompatible with freedom of expression. In the philosophical and legal traditions on freedom of expression, viewpoint-discriminatory restrictions on speech are very difficult to justify. Under American free speech jurisprudence, they are almost impossible to justify, as they trigger ‘strict scrutiny’—the most demanding test in U.S. constitutional law.^17^ In other legal traditions, where freedom of expression is a qualified right routinely balanced against other rights, such restrictions are easier to justify (as we will soon see)—but they still require justification.

It is deeply plausible, then, that demotion can raise free speech concerns.^18^ This is true, anyway, when demotion is *state-compelled*. Insofar as new regulation may well involve coercing or incentivizing platforms to demote content, we have powerful reason to inquire into whether such policies can be justified at the bar of free speech; and much of our analysis below will be relevant to such evaluations.^19^ But now suppose that platforms demote certain content *voluntarily*. Consider, for example, Meta’s policy of demoting speech promoting falsehoods about climate change.^20^ Such a demotion policy is viewpoint-discriminatory; after all, it leaves untouched speech promoting other points of view about climate change. Such a policy is itself based on a substantive judgment that the viewpoint expressed is false, harmful, or otherwise misguided, such that it is desirable for fewer people to encounter and be persuaded by it, or to see it less frequently. Does such *voluntary* viewpoint-based demotion need to be justified at the bar of free speech?

One’s answer to this question will not be linked to demotion in particular; it will flow from one’s general view on whether platforms have moral or legal duties to respect free speech. In the U.S., platforms do not have any *legal* duties to respect free speech; the First Amendment binds state actors (and other entities acting in a state-like capacity), not private corporations. The US Supreme Court has ruled that platforms’ *own free speech rights* mean that they enjoy broad latitude to amplify or demote, allow or remove, whatever content they like.^21^ They are legally free to undertake whatever content or viewpoint discrimination they want.^22^ But it is perfectly coherent to think that, even if platforms have no enforceable *legal* duty to respect free speech, they may have a *moral* duty.^23^

The claim that private platforms have *moral* duties to respect the free speech of their users has two potential sources. First, social media networks have become such central forums for public discourse that they have a duty to respect free speech *as if* they were run by states.^24^ Even if you doubt that, there is a second potential source: at least some platforms have arguably acquired the duty *voluntarily*. The largest social media networks have committed themselves to a public self-conception as forums for open discourse.^25^ Their own declarations have invited this ethical scrutiny.

In fact, some platforms embrace explicitly the claim that these moral responsibilities simultaneously qualify as legal (or at least *quasi*-legal) responsibilities under international human rights law (unenforceable though they may be). The UN Guiding Principles on Business and Human Rights set out the responsibilities of corporations to respect human rights.^26^ Adopting these principles enables companies, who eschew public criticism, to point to external standards as the basis of their decisions. To be sure, what exactly these responsibilities require in practice is controversial; and the international legal responsibilities of corporations at issue are not currently enforceable by any authoritative judicial body. Even so, the idea that *international human rights norms* apply to content moderation decisions by platforms is quickly becoming mainstream. For example, Meta’s Oversight Board embraces human rights norms as the basis of its decisions. The Board has the authority, contractually agreed with Meta, to review Meta’s content decisions on Facebook, Instagram, and Threads (upholding or striking them down in individual cases). In doing so, it explicitly appeals to the normative tests for restricting the right to freedom of expression set out in Article 19 of the International Covenant on Civil and Political Rights (ICCPR).

As we will see momentarily, these tests closely mirror mainstream philosophical views on the permissibility of imposing harm or otherwise interfering with agents’ rights. Thus even readers who doubt that businesses have *bona fide* legal responsibilities under international human rights law can accept (1) that platforms have *moral* duties to respect free speech, and (2) that normative insights from human rights law plausibly inform our philosophical reasoning about what those duties demand. Admittedly, human rights norms do not straightforwardly produce obvious answers to the difficult normative questions arising in this space.^27^ They do, however, offer a propitious basis from which to begin our normative reasoning about content moderation.^28^ Indeed, the Oversight Board has flagged *demotion* as a form of interference with freedom of expression (even when done voluntarily by platforms) that stands in need of justification under these principles:

The Board is concerned about Meta’s practice of demoting content that third-party fact-checkers rate as ‘false’ or ‘altered’ without informing users or providing appeal mechanisms. *Demoting content has significant negative impacts on freedom of expression*.^29^

Instructively, the decisionmakers most keen to figure out what to do about demotion policy are those *already* inclined to deploy an IHRL approach. A further payoff of our argument, then, is to use demotions policy to illustrate how human rights principles can provide fruitful normative guidance for platform policy.

### Justifying Speech Restrictions Through International Human Rights Norms

In keeping with the approach followed by the Oversight Board and its intellectual defenders, we will deploy normative insights from international human rights law to evaluate platforms’ demotion policies. Under Article 19 of the ICCPR, interferences with the right to freedom of expression must pass several tests to be justified. First, *legitimacy* requires that restrictions serve some recognized goal. Second, *legality* requires that restrictions be clearly set out in keeping with the requirements of the rule of law (such as setting out rules in advance and not applying rules retroactively). Third, *suitability* requires that restrictions be suited to achieve their goal. Fourth, *necessity* requires that restrictions represent the least intrusive means available. And fifth, *proportionality stricto sensu* requires that the benefits produced by the restriction be sufficient to offset costs.^30^

These core principles determine the permissibility and impermissibility of measures interfering with rights under human rights law—whether imposed by states or corporations. These requirements embody a decisional framework developed by Robert Alexy and widely adopted by constitutional democracies’ human rights law.^31^

Crucially, it is worth observing that the same basic principles arise, in similar form, in the non-consequentialist moral and political philosophy of rights. Consider, for example, the use of force by states or persons in self-defence, or defence of others. Political philosophers have for centuries theorized the legitimate aims of state power—with the liberal tradition emphasizing the fundamental role in protecting people from attacks on their basic rights and liberties.^32^ Likewise, legal philosophers have offered painstaking analysis of the moral value of legality.^33^ And moral and political philosophers have offered elaborate explorations of the demands of necessity and proportionality—moral principles that offer substantial illumination in the context of speech governance.^34^

These norms are now being used to offer guidance to the content moderation context. How do they work in practice? Here is an easy hypothetical. Suppose platforms were to decide that the right way to stop electoral misinformation was to ban *all speech about elections*. Assume that such a goal is legitimate, that it is suitable (in that it would be oriented to catching the relevant harmful speech), and that it is compatible with the value of legality (since the policy would be publicly set out in advance and would be applied even-handedly). Even so, the policy would be both unnecessary and disproportionate: unnecessary because it is not necessary to ban all such speech to catch the harmful speech when there are equally effective and less restrictive measures available; and disproportionate because the costs of such a rule (a huge amount of valuable discourse and information-sharing suppressed) are too great to justify its benefits (the limitation of a much smaller amount of genuinely harmful speech). The preferred remedy, of course, is to craft a narrowly tailored rule disallowing baseless electoral misinformation, especially during the run-up to an election.

That example involves a hypothetical removal policy, but there is no reason why such reasoning cannot also be applied to demotion policy. In the next section, we briefly discuss legitimacy, legality, and suitability. We then turn to the two tests that we think will usually be determinative in enjoining demotion over removal: necessity and proportionality *stricto sensu*.

## Demotion: Legitimacy, Legality, and Suitability

### Legitimacy

Consider *legitimacy*, which concerns the *reasons* for moderating content. Under IHRL, limitations to freedom of expression are only legitimate when they seek to further the protection of other recognised interests.^35^ How could demotion pass the legitimacy test? Here we focus on one particular goal: preventing harm.^36^ More precisely, we focus on the use of demotion to defend people (offline and online) from risks of harm that online content poses—something platforms, we assume, have a moral duty to strive to do.^37^ This is the dominant rationale that justifies the removal of content—whether incitement to violence or racist abuse or vaccine misinformation. That same rationale, we suggest, can justify the demotion of content.^38^

Perhaps there are other legitimate rationales for demotion. One interesting question is whether companies’ reasons for interfering with speech is restricted to the same list applying to states under the ICCPR, or whether they may invoke a broader range of interests, such as their own business interests. For example, consider Meta’s policy of demoting ‘low-quality comments’ or ‘links to websites requesting unnecessary user data’; it seems that the rationale here is simply to improve the user experience, rather than prevent harm. We grant the possibility of other legitimate rationales for demotion.^39^ But harm prevention is the one we shall discuss here.

### Legality

Next, consider *legality*, which requires rules to be spelled out precisely in advance. One demand of legality is *transparency*, such that users have easy access to information about what the demotion policy is and how it is applied, so that they can reasonably predict how their content will be treated. At present, we suspect that demotion policies are not sufficiently transparent to satisfy legality, in multiple respects. For starters, they are worded very vaguely, such that users have little basis for predicting when their speech will be demoted. Further, those whose speech is demoted are typically not *notified* of this fact. Finally, we lack information on how well policies are (or aren’t enforced)—a massive can of worms, with which European regulators will have to contend. This opacity is especially problematic for demotion, which, unlike a clear removal, is often invisible to the user, making it impossible for them to know their speech has been restricted.^40^

Academics and civil society alike have long identified transparency as a cornerstone of platform accountability. Efforts like the Santa Clara Principles explicitly call for clarity on content moderation measures beyond simple removal.^41^ Yet, scholars rightly caution against treating transparency as a silver bullet. They note that ‘transparency’ can be an ambiguous concept, easily co-opted by platforms into performative reporting that offers the illusion of oversight without ceding real power.^42^ Scholars have even questioned its intrinsic value, arguing that it can become a form of surveillance.^43^ These critiques underscore the need to move beyond performative gestures and toward what has been termed ‘meaningful transparency’^44^ or ‘platform observability’^45^ which are robust enough to satisfy the legality requirement.

Another related demand of legality concerns *fairness*—specifically, even-handedness in the enforcement of rules. It is by now ubiquitous for people who think their content is being demoted aggressively to complain of being ‘shadowbanned’. For example, some have argued that content promoting Palestinian rights is being demoted in the context of the most recent Israel-Hamas war.^46^ Greater transparency will also be useful in responding to these kinds of worries.^47^

Our central claim is that legality requires platforms to be explicit about *what* content they demote, *why* they demote it, and *how* they reach those decisions. We suggest that first, at the policy level, platforms must explicitly define the categories of content subject to demotion with clear examples. Second, at the individual level, users must be notified when their content is demoted, with a clear explanation of the policy violated. Finally, at the systemic level, platforms must publish regular, detailed data on the aggregate volume and error rates of demotion (mirroring existing reports on content removals).^48^ This data, importantly, should be accessible to regulators, independent researchers, as well as a wider range of actors that comprise the content moderation ecosystem – including bodies such as Meta’s Oversight Body and out-of-court dispute settlement bodies. This multi-stakeholder approach, which provides clarity to users, auditors, and the public, is already being codified in laws like the EU’s Digital Services Act, which grants users a right to complain about visibility restrictions and seek redress through oversight bodies (Articles 20 and 21, DSA).^49^

One particularly fraught area in need of reform, at the bar of legality, concerns so-called *borderline content*—i.e., speech that comes close to violating their rules, but falls short.^50^ As mentioned, Meta has a policy of demoting such content. Consider: What, exactly, is the content borderline to hate speech? Does it encompass legitimate discussions of divisive topics like race and gender identity? Users should be empowered to understand when demotion is likely to occur. Borderline speech is especially opaque, relative to other categories of demoted content, in light of how it is seemingly operationalized. As far as we can tell, content is deemed borderline when the AI classifiers judge it to have some probability of constituting violating content, whereby the probability falls short of the threshold required for removal.^51^ (So, maybe the classifier requires 90% confidence that a post is hate speech for it to be removed, and borderline content concerns posts that fall within the 80-89% range). A superior approach, *vis a vis* legality, would be to designate clearly, for each category of violating speech, what counts as the relevant borderline speech that is to be demoted. (For example, we might think that ‘borderline hate speech’ could include speech that involves racist stereotypes, but none of the exact negative stereotypes that run afoul of the hate speech rules.) Ideally, platforms would deploy dedicated AI classifiers for each category of appropriately demoted content, rather than piggybacking off of existing classifiers targeting removed content—in other words, removing certain content based on a judgment that *it* is appropriately demoted, not on a lukewarm probability score based on its similarity to removable content. This would ensure greater guidance to users on what exactly gets demoted—precisely what legality demands.

We surmise that platforms’ demotion practices would likely flunk the legality test. However, in order to progress the analysis, let’s assume that the goal being pursued by demotion is legitimate and that the measure complies with the legality requirement. When, then, could platforms justifiably demote content without unjustifiably interfering with the right to freedom of expression? The remaining tests are suitability, necessity, and proportionality *stricto sensu*; we will briefly discuss suitability before turning to the final two, on which we will dwell at length.

### Suitability

*Suitability* requires that interference with the right to freedom of expression must actually contribute to the realisation of the legitimate objective—that is, the measure and the goal being pursued need to be rationally connected.^52^ For instance, restricting speech that undermines public health may be justified if it actually stands a reasonable chance of contributing to the protection of public health. A law banning the speech of the government's political opponents under the pretext of promoting public health would flunk this test. Suitability helps to catch measures that stand no plausible chance of achieving their putative objection—and perhaps may have been motivated by other, illegitimate goals. The analogue requirement in the philosophical literature is that some use of force or imposition of harm must have a reasonable chance of success or effectiveness to be justified (though philosophers have tended to endorse a stronger version of this than lawyers, perhaps out of lawyers’ recognition that judges will often be ill-equipped to make anything more than a minimal assessment of the relevant empirical claims).^53^

The suitability test is sensitive to the goal of the measure under scrutiny. We have noted that the goal of demotion, in an important range of cases, will be to prevent harm that arises when audiences are exposed to certain content in large doses (an idea to be explored further momentarily). The question, then, is whether demotion is the kind of policy that is oriented to achieving that particular goal, such that it has at least minimal prospects for success. Almost by definition, we submit that it does: to demote content *just is* to reduce exposure.^54^

The contrast between demotion and other measures helps to clarify this point. Suppose the goal was something else—such as empowering users to avoid (or better anticipate) sensitive content (like images of graphic violence). In such a case, the obvious suitable remedy would be an interstitial ‘click-through’ warning screen, empowering users to decide for themselves whether to see it. Or suppose the goal was to prevent harm that certain content causes to children, but doesn’t cause to adults. In that case, the most suitable remedy would be to ‘age-gate’ the content so that minors were not exposed to it whatsoever. In neither case would demotion be the suitable remedy.

In contrast, demotion’s suitability becomes clear when the objective is to reduce a type of content’s overall prevalence, rather than just targeting specific audiences. For example, individuals seeking self-harm content are the most likely to click through a warning screen, defeating the policy’s purpose. The friction of a ‘click-through’ screen, therefore, fails to reduce exposure for those most at risk. This type of remedy is also unsuitable if the goal is to protect people *outside* the platform. Consider falsehoods spread about a particular community, which could spark hostility or even violence against its members. People vulnerable to such attacks would not necessarily be directly exposed to the harmful content or a warning screen, rendering those remedies ineffective. Demotion, however, effectively reduces the content’s overall reach and, therefore, meets the suitability test.

## Demotion and Necessity

The analysis then requires us to move into the next step and assess *necessity*: platforms should suppress speech no more than necessary to achieve the relevant goal of preventing harm, all other things equal.^55^ Just as police should use the least amount of force necessary to disarm an aggressor, and legislators should impose the lowest amount of cost on citizens necessary to achieve their policy objective, platforms too should not engage in unnecessary levels of content moderation.^56^ Indeed the Oversight Board has itself invoked necessity as potential grounds for opting for an alternative remedy instead of removal:

The Board believes that, where possible, Facebook should use *less restrictive measures to address potentially harmful speech* and protect the rights of others before resorting to content removal and account restriction. At a minimum, this would mean *developing effective mechanisms to avoid amplifying speech* that poses risks of imminent violence, discrimination, or other lawless action, where possible and proportionate, *rather than banning the speech outright* (Oversight Board, case decision 2021-001-FB-FBR, p. 29, emphasis added).^57^

But in what kinds of cases does removal fail the *necessity* test, such that demotion is preferable?

Our answer turns on a distinction between two different kinds of harmful content. The rough distinction is between content that is harmful when encountered in small doses, on the one hand, and content that only becomes harmful when encountered in large doses, on the other. Here ‘content’ can refer to individual posts (e.g., a single post might get amplified to a massive audience), but more typically it refers to *aggregations* of similar content.

The claim that some content is harmful only in high doses is perfectly familiar in other contexts. A small amount of carbon in the atmosphere doesn’t cause harm. But a large amount of carbon clearly does. Likewise, a small amount of contaminants in a river (e.g., human waste) does not pose a serious problem; yet large amounts can pose risks to the wildlife or swimmers’ health. Sonically, low decibel sound poses no risk to the inner ear drum, but above some decibel threshold sound causes damage.

But how might such a distinction arise in the context of harms caused through speech? What sort of content is harmful even without amplification, and what sort of content requires amplification to be harmful?

Consider content that can lead to self-harm. One diabolically persuasive post encouraging someone to kill herself, and offering up precise instructions on how to do so, risks harm, and is intuitively unacceptable. This content is *individually* dangerous. In contrast, consider content that promotes unhealthy body image stereotypes—e.g., photos of very slim models. When an impressionable user encounters such content in low doses, it is unlikely to cause harm. But when the user finds herself *bombarded* with such content—encountering it with great frequency—at some point the content indeed becomes harmful.^58^

Or consider again so-called borderline speech (speech that comes close to violating content rules, but doesn’t cross the line). As we noted before, Meta has a policy of demoting such speech. Consider borderline hate speech—speech that comes close to violating the outright ban on hate speech, but doesn’t cross the line.^59^ Racially insensitive jokes that turn on negative stereotypes potentially fall into this category.^60^ As the literature on so-called ‘microaggressions’ explains, this kind of content—like pin pricks—is reasonably innocuous in low doses, but can become an exhausting assault on one’s sense of identity or belonging in high doses.^61^ In these cases*, what matters is preventing accumulations of this content from flooding the feeds of particular people who would be harmed by it*. Demotion’s goal is to ensure that impressionable individuals don’t see *too much* of this content.^62^ Here the analogy with turning down volume to protect an individual’s inner ear drum is apt.

But in other cases, the chief purpose of demoting content is to *reduce its dissemination through the population as a whole*.^63^ Consider misinformation suggesting that climate change is a hoax. In small doses, such misinformation is unlikely to cause harm. Only when amplified will such posts risk harm. By inducing false beliefs about climate change, such content makes it more likely that the relevant collective agents (such as states) will fail in their moral duties. Future harms to individuals (from flooding, famine, and so on) flow from that collective failure. So what needs to be prevented, in the first instance, is the formation and maintenance of false beliefs about climate change. But in low doses, it is unlikely that climate misinformation is going to induce sufficiently widespread false beliefs.^64^ Why? Collectively, we enjoy epistemic defensive systems that help us to process bad information; indeed for Millian reasons, some quantity of falsehood may even strengthen our collective systems.^65^ Yet in high doses, when flooded with falsehood, these systems can be overwhelmed. Of course, even when content isn’t amplified, particular individuals may still become taken by it. But it is far less likely that the relevant *group agents* will be taken by it. With harms like climate change—perpetrated via public policy failures—that are a matter of collective action, it is *group agents* (such as states) who have the most significant duties to act in certain ways to prevent or redress harm. These agents are plausibly relatively unaffected by low doses of misinformation, but can be affected by high doses.

In the absence of content moderation interventions, climate misinformation likely gets algorithmically amplified, in virtue of its tendency to provoke outrage and strongly felt sentiments.^66^ Yet by arresting such amplification through demotion, platforms can bring down the dosage of this content, such that it is no longer harmful. If that empirical conjecture is correct, the normative implication is clear: removal is unnecessary to prevent the harm.

Plainly, not all misinformation can adequately be handled through demotion. Consider misinformation contending that drinking bleach cures Covid-19 (a claim currently banned by Meta). Such speech risks serious harm to gullible users exposed to such speech; it is for this reason that such speech is unacceptable. This is so, it seems to us, even when it is encountered in a low dosage. To be sure, all speech that is harmful in low doses will be *even more harmful* in large doses. If the platform were overrun with such speech, this would aggravate its danger substantially. Accordingly, the amplification of such speech through engagement-optimization algorithms would certainly increase the risk of harm it poses. Our point is simply that, even without such amplification, this speech poses an unacceptable risk of harm. Outright removal is, then, *necessary* to adequately confront its danger.^67^ But this simply isn’t true for climate misinformation. Some speech causes or risks harm only in larger doses, such that, in small doses, it is innocuous. For this content, removal is *unnecessary*, making a less costly intervention—demotion—preferable.

Consider one further example. Recall again Meta’s policy of demoting borderline content. It’s not clear what exactly is encompassed by, for example, borderline incitement. But it could plausibly involve militant rhetoric that vilifies those who belong to an opposing political party.^68^ Speculatively, in high doses such content causes harm by altering the norms of discourse*—*eroding norms of respect or civic friendship in how citizens who disagree address one another, coarsening public discourse and exacerbating polarisation. When such speech besieges our public sphere, it may cause us to relate to one another less as fellow citizens who disagree, and more as enemies. That, in turn, may make it less likely for relevant group agents—like political parties—to cooperate with one another. Yet in low doses, something that people encounter occasionally, it’s plausibly unproblematic. While once again this is an empirical conjecture, if it’s correct it could justify the normative thesis that removing borderline incitement is unnecessary, rendering demotion a more apt remedy. For these sorts of content, the necessity test militates in favor of demoting such content, rather than removing it.

Our claim in this section—that certain speech becomes untenable only in large doses—is emphatically a *hypothesis*, which can only be corroborated with empirical work in social science testing its plausibility. But it is intuitively plausible. While it remains to be seen the extent to which these specific empirical claims (e.g., about the effects of militaristic discourse) are true, our central claim is not empirical but normative: for speech that becomes harmful only in large doses, demotion is the right remedy. If demotion can bring the dosage under the relevant threshold that it qualifies as innocuous, then outright removal is unnecessary, and so objectionable.

Where, exactly, is that threshold? It is very difficult to say; we doubt that platforms are in position to answer the ‘how *much* demotion?’ question adequately. Such a threshold will be difficult to pin down with precision in practice. It will be very fraught to try to determine a precise number of views for a piece of content (or category of content) beyond which that content’s further distribution becomes harmful. Consider: How much content depicting unhealthily thin body images to teenage girls, or questionable diets, will tip them into an eating disorder? How many racist jokes will induce a sense of inferiority in someone exposed to them? How much climate change misinformation will inhibit the possibility of constructive discussion on this topic? The matter is complicated further by the fact that different populations will have different susceptibility levels. We believe much more empirical evidence is necessary to get firm traction on these questions; indeed, one aim of this article is to provide the normative case for motivating precisely such empirical inquiry.^69^

Because of the difficulty addressing the threshold question, and the absence of adequate empirical evidence as to the exact value of each relevant threshold, it is not reasonable to expect platforms to calibrate their levels of demotion so that the level precisely tracks the potentially-objective-but-currently-unknown threshold (supposing one exists for each category). So what level of demotion should platforms adopt? We suggest that the default demotion setting for problematic content should involve (1) excluding the content from recommendations, and (2) lowering the place of content within search results. Such a default policy plausibly strikes the balance in preventing such content from being amplified to users; yet those particularly keen to find such content will still be permitted to do so. This isn’t necessarily the perfect demotion setting for all categories, to be sure; but it is a reasonable default option, which is plausible in the midst of uncertainty as to what thresholds for harm might be. It’s reasonable because wherever the threshold is, the default setting is likely to bring the dosage of harmful content below it.

We have argued that demotion is preferable to removal in cases where removal is unnecessary to prevent harm; demotion is adequate to prevent harm. But now consider an objection. Take the example of content promoting extreme weight loss strategies. Some people would be harmed by this content in virtue of its algorithmic recommendation; but if this content gets demoted, these people will be spared harm. So far, so good. However, there is another category of people who will nevertheless seek out such content and find it. Even if such content is excluded from recommendations and substantially lowered in search results (per the default demotion setting discussed above), harm may still befall these people, in light of their relentless search for such content. Isn’t there an important sense, then, in which removal is necessary for that population?^70^

In reply, we can grant there will be cases in which removal is necessary to protect one population but not another. Children are a case in point; perhaps it is defensible to age-gate extreme weightloss tips, so that this content is effectively banned for children but not for adults. But set that point aside; we’ll assume the objection here concerns an adult population, within which certain people will be adequately protected via demotion but other people won’t be. If a policy of removing content is necessary to protect the more vulnerable population, is such a policy therefore justified with regard to the entire population?

Not necessarily. Passing the necessity test is necessary, but it’s not sufficient; we’ve still got one condition left: proportionality. Our own judgment is that it would be disproportionate to ban someone from posting weight loss content. For example, a post describing how to do intermittent fasting, for example, is appropriately demoted, but it should not be banned; to ban such a post would involve a disproportionate burden to speakers’ and audiences’ liberties, denying them any opportunity to express or access such content on the platform. What, exactly, is involved in making such proportionality judgments? We turn to that issue now.

## Demotion and Strict Proportionality

The final hurdle that any speech restriction must clear involves a strict proportionality test. The question is whether the benefits of some measure restricting speech—such as preventing harm—can justify the costs—namely, impact on users’ free expression interests.

Our point here is relatively straightforward: demotion tends to impose fewer absolute costs than removal does. As a result, a proposed demotion policy is more likely to clear the strict proportionality hurdle than removal is, for the following reasons. When speech is demoted, speakers remain free to express themselves. Freedom of speech is partly justified with reference to speakers’ autonomy interests in expressing themselves; this requires *some* access to an audience. But the audience need not be vast. As Erin Miller has argued, our autonomy interests do not require that our speech enjoy *mass amplification*; accordingly, she thinks, regulation of such amplification processes does not necessarily affront freedom of speech.^71^

The upshot is that speakers’ autonomy interests can be satisfied even without mass amplification of their speech. Accordingly, milder forms of demotion may leave them with sufficient audience exposure, such that their autonomy interests remain fully satisfied. If that is right, then when we’re pricing the *costs* of a demotion policy for the purpose of proportionality analysis, these costs are simply lower than the costs involved with removal. To be sure, this is a matter of degree. The most sweeping forms of demotion may make it virtually impossible for audiences to encounter or discover what a speaker says, even if they’re looking for it.^72^ Below some threshold, speakers have insufficient audience access to satisfy fully their expressive interests. Still, they will be able to satisfy them to *some* degree, making demotion preferable to outright removal in this respect. This is especially so once we factor in the contingent fact that removal policies often then lead to temporary or permanent suspensions of one’s account, or other penalties (including ‘probationary’ measures such as the demotion of *all* content one posts). Thus even when demotion is severe, it remains less costly for speakers than outright removal.

The same point applies to listener interests. One of the central justifications for freedom of speech appeals to the interests of audiences to hear many points of view, so that they can make up their own minds. These interests are impacted by demotion; but again, with many forms of demotion, listeners remain free to locate content they want to see—by going to the accounts of people they follow, or searching with especial assiduousness. In contrast, when speech is removed entirely, audiences cannot see it, even if they look hard for it. Accordingly, when pricing the cost of demotion for audiences, it will be lower than removal.

The final respect in which demotion imposes fewer costs than removal concerns the *collateral* consequences of content moderation. As is widely recognized, content moderation is a deeply fallible process; it regularly produces false positives, taking down legitimate content that ought to have remained up.^73^ But when demotion goes wrong and produces false positives, these are simply less egregious than when removal goes wrong and produces false positives. Again, that’s especially so when we consider that accounts which get demoted don’t receive strikes counting toward a suspension.

Taken together these points simply make it easier for a transparent demotion policy to survive a proportionality cost-benefit analysis than a removal policy, all other things equal.^74^

Now consider the practical implications of this point. Consider once more Meta’s policy of demoting borderline content. We already argued that removing this content would potentially flunk necessity. But it would also flunk proportionality. In these kinds of cases, a plausible thought is that the speech causes *some* level of harm; it simply isn’t enough harm to justify *removal* as a sanction. Removal would be disproportionate to the gravity of the harm posed by the content. In contrast, demotion potentially *is* proportionate.

We say *potentially* because it all depends on exactly how the demotion policy is operationalised. If the relevant classifier is too broad—demoting *all* mental health discussion by deeming it ‘borderline’ to self-harm advocacy, or demoting *all* discussion of race by deeming it ‘borderline’ to hate speech—then such a policy will remain disproportionate. As we noted in our discussion of legality, Meta simply has not given us enough information about its borderline content demotion policies in order to adjudicate whether they meet the proportionality test. Our point is simply that, in principle, it’s plausible that it could meet that test, provided it was drawn narrowly.

Before concluding, one theoretical point is in order. We have been dealing with the proportionality cost-benefit analysis in roughly consequentialist terms, such that the task is simply one of comparing benefits and costs. In legal scholarship, this has been a frequent object of discussion, with scholars arguing that rights cannot be measured and therefore there is no common metric for balancing opposing legal values.^75^ Elsewhere one of us has defended the claim that the appropriate proportionality analysis for rights-interfering-activity (such as speech governance) should incorporate deontological distinctions between narrow and wide proportionality, as developed in the philosophy of self-defense and war.^76^ We have skated over these details, since they are unnecessary for present purposes. But our insight in this section can be easily expressed in these terms. So, take narrow proportionality, which is a matter of whether a speaker is *liable* to bear the costs of a particular (defensive or punitive) sanction—paradigmatically, because through his past, ongoing, or imminent wrongdoing he has made himself *liable* to bear those costs. The point is that, for mild harms, the speaker may have made himself liable to *demotion*; but he is not liable for *removal*. He has indeed breached a duty he owes to others—but it is not a particularly weighty duty, and removal would accordingly be an excessive response to its breach.

There are further proportionality concerns that we have not addressed—specifically, concerning further collateral costs of demotion policies.^77^ Part of this depends on how the demotion policies are themselves specified. If speech deemed ‘borderline’ to self-harm advocacy is very broad, such that *any* discussion of mental health is encompassed, it plainly has *massive* collateral costs to demote such content.^78^ We doubt such a far-reaching demotion policy could be justified at the bar of proportionality. Just as removal policies can have disproportionate effects on already marginalised groups,^79^ so can demotion policies. For example, demoting borderline nudity content clearly generates negative impacts on sex workers, with studies showing that the practice makes it harder for them to find and share strategies for staying safe.^80^ Similarly, platforms like Instagram have been criticized for demoting posts depicting women’s bodies, nudity, and sexuality. This disproportionately harms women artists and activists, hindering their ability to reach new audiences and express themselves legitimately.^81^ Research has also shown that TikTok’s demotion of content from disabled, queer, and trans creators restricts their access to crucial education, advocacy, and community-building resources.^82^ Taken together, these examples raise serious questions about whether such demotion can be justified unless measures are taken to reduce considerably these collateral costs.

Moreover, while we have focused on collateral costs for free speech, there are also collateral financial concerns (e.g., suppose influencers’ and other online businesses’ legitimate speech were demoted as collateral damage thanks to an overbroad demotion rule, causing them substantial financial setbacks).^83^ One upshot is that platforms ought to design demotion policies in ways that minimize this collateral damage. It is implausible to think that they will ever *eliminate* these collateral costs; even if we agreed on the ideal system of speech rules, current AI technology—a crucial tool deployed by the largest platforms to manage the sheer volume of user-generated content—simply cannot implement those rules with perfection.^84^ The less collateral damage there is, and the less it disproportionately burdens particular groups, the more tolerable it will be.

## Conclusion

The aim of this article has been to contribute to the burgeoning conversation on the normatively appropriate role of demotion in the governance of speech. In making our argument, one core aim has been to zoom in on how, exactly, demotion policy can illuminate broader questions about how to apply international human rights norms to the distinctive context of online content moderation.

We have argued that demotion, whereby platforms select a category of content for general reduced visibility, stands in need of moral justification just as removal policies do. But that justification can be found. We argued that in cases where content causes harm only when it is aggregated and amplified with similar content, outright removal is often unnecessary to defuse the harm. In other cases, demotion is apt because (unlike removal) it strikes a proportionate balance between benefits and costs, e.g. enabling speakers to express themselves even as the reach of their speech is reduced. We applied these insights to a range of controversial content areas, including misinformation, hate speech, self-harm content, and borderline content.

The public discourse on content moderation is often a pitched battle between those who decry censorship and want fewer restrictions on content, and those who think the demands of harm prevention require greater action. A virtue of demotion is precisely that it attends to the normative impulses underpinning both views in this debate.^85^

## Footnotes

1 Tarleton Gillespie, ‘Do Not Recommend? Reduction as a Form of Content Moderation’ (2022) 8 Social Media + Society 205630512211175.

2 Meta Transparency Center, 2023, ‘Types of Content We Demote’, available at https://transparency.fb.com/features/approach-to-ranking/types-of-content-we-demote/ (accessed 2 July 2025)

3 TikTok For You feed Eligibility Standards, 2024, available at https://www.tiktok.com/community-guidelines/en/fyf-standards (accessed 10 July 2024)

4 X, Sensitive Media Label Review Update, available at https://help.x.com/en/rules-and-policies/sensitive-media-label-not-removed (accessed 2 July 2025) See Appendix 1 for further summaries of these policies.

5 See Gillespie (n 1); Paddy Leerssen, ‘An End to Shadow Banning? Transparency Rights in the Digital Services Act between Content Moderation and Curation’ (2023) 48 Computer Law & Security Review 105790; Eric Goldman, ‘Content Moderation Remedies’ (2021) 28 Michigan Technology Law Review 1.

6 For legal and philosophical discussions that are indirectly related to the issue of demotion, see Erin L Miller, ‘Amplified Speech’ (2021) 43 Cardozo Law Review 1; Seth Lazar, *Connected By Code: How AI Structures, and Governs, the Ways We Relate* (Oxford University Press, forthcoming).

7 Daphne Keller, ‘Amplification and Its Discontents: Why Regulating the Reach of Online Content Is Hard’ (2021) 1 Journal of Free Speech Law 227.

8 There are other ways to reduce the visibility of content, such as interstitial warning screens requiring users to ‘click through’ to see content (e.g., when a video contains graphic violence). But the purpose of interstitial warning screens is not to reduce visibility, but rather to empower users to decide for themselves whether to see it. For this reason, we think the appropriate use of this technique requires a separate analysis.

9 See Leerssen (n 5).

10 Gillespie (n 1) 4.

11 Leerssen (n 5). This is not to deny that demotion policies will be algorithmically encoded so that AI-powered curation systems will be automatically applying classifiers to determine what gets demoted.

12 See Gillespie (n 1); Goldman (n 5).

13 See Michael D Ekstrand and Martijn C Willemsen, ‘Behaviorism Is Not Enough: Better Recommendations through Listening to Users’, *Proceedings of the 10th ACM Conference on Recommender Systems* (ACM 2016); Silvia Milano, Mariarosaria Taddeo and Luciano Floridi, ‘Ethical Aspects of Multi-Stakeholder Recommendation Systems’ (2021) 37 The Information Society 35; Jonathan Stray, ‘Designing Recommender Systems to Depolarize’ [2022] First Monday; Lazar (n 6).

14 For a review of the relevant points, see Leerssen (n 5).

15 Renee DiResta, ‘Free Speech Is Not the Same As Free Reach’ (*Wired*, 28 August 2018) <https://www.wired.com/story/free-speech-is-not-the-same-as-free-reach/>.

16 For relevant discussion, see Michael Coenen, ‘Of Speech and Sanctions: Toward a Penalty-Sensitive Approach to the First Amendment’ (2012) 112 Columbia Law Review 991.

17 Even when not viewpoint-based, demotion policies are often content-based and presumptively unjustified (though the presumption is easier to rebut than for viewpoint-based restrictions). For classic discussion, see Geoffrey R Stone, ‘Content Regulation and the First Amendment’ (1983) 25 William & Mary Law Review 189.

18 The claim that demotion raises the same basic free speech issues as removal is central to the argument in Keller (n 8). See also JP Messina, *Private Censorship* (Oxford University Press 2024) 118.

19 For example, the Digital Services Act in the EU requires ‘very large online platforms’, i.e. those with more than 48 million active monthly users, to undertake risk assessments, and formulate enforceable plans for how to mitigate risk—plans that could involve demoting certain content.

20 See https://sustainability.atmeta.com/blog/2022/11/04/our-approach-to-climate-content/. Note that Meta no longer demotes fact-checked misinformation in the United States, but continues to do so in the rest of the globe.

21 Moody v. NetChoice, LLC and NetChoice, LLC v. Paxton, 603 U.S. 707 (2024). That position is compatible with their possession of legal obligations to remove certain unlawful speech.

22 Such editorial discretion for social media networks also exists in the UK and EU, though the matter is more ambiguous. There are *some* vague legal requirements on platforms to respect free speech in the EU and UK, but it remains highly unclear what their force will be. For example, EU’s Digital Services Act holds that ‘very large online platforms’ have duties to ‘pay due regard’ to freedom of expression. In the UK, the Online Safety Act 2023 establishes that Category 1 platforms, i.e. the largest and riskier user-to-user services, have ‘a duty to have particular regard to the importance of protecting users’ right to freedom of expression within the law’ (Section 22(2)).

23 Similarly, traditional media organizations may be properly immune from state interference with their editorial decisions; yet it is still plausible that they have ethical responsibilities to make their editorial decisions in certain ways rather than others.

24 See Matthew Kramer, *Freedom of Expression as Self-Restraint* Oxford: Oxford University Press, 2021), 58-59. For critical reflection on this family of arguments, see Messina, *Private Censorship,* 123ff.

25 For example, see Meta’s statements on voice: https://about.meta.com/uk/actions/promoting-safety-and-expression/. Many other public statements reiterate this point; e.g., Meta’s President for Global Affairs Nick Clegg recently noted: ‘We know that when enforcing our policies, our error rates are still too high, *which gets in the way of the free expression that we set out to enable*’ (Alex Heath, ‘Meta says it’s mistakenly moderating too much speech’, *The Verge* Dec 3, 2024, URL: https://www.theverge.com/2024/12/3/24311513/meta-content-moderation-mistakes-nick-clegg?ref=everythinginmoderation.co/ (emphasis added)). Similarly, Elon Musk, the owner of X, has committed to defending ‘free speech’ on his platform: https://x.com/elonmusk/status/1519036983137509376.

26 See Evelyn M Aswad, ‘The Future of Freedom of Expression Online’ (2018) 17 Duke Law & Technology Review 39–41.

27 See Douek for criticism of the use of IHRL in content moderation. Evelyn Douek, ‘The Limits of International Law in Content Moderation’ (2021) 6 UC Irvine Journal of International, Transnational, and Comparative Law 37.

28 For relevant discussion, from which we have benefitted enormously, see Aswad (n 26); David Kaye, ‘Report of the Special Rapporteur on the Promotion and Protection of the Right to Freedom of Opinion and Expression’ (UN Human Rights Council 2018) A/HRC/38/35.

29 Oversight Board case 2023-029-FB-UA, Altered Video of President Biden (emphasis added).

30 In the historical commentary, the final three tests were subsumed into a ‘necessity and proportionality’ test that has since been unpacked into the three components. UN Human Rights Committee, ‘General Comment No. 34 - International Covenant on Civil and Political Rights’ (United Nations 2011) CCPR/C/GC/34 para 22. Even to this day, some scholars and lawyers will use the term ‘proportionality’ to refer to the final three tests.

31 See Robert Alexy, *A Theory of Constitutional Rights* (Oxford University Press 2002). For a discussion of the influence of Alexy's framework, see Kai Möller, *The Global Model of Constitutional Rights* (Oxford University Press 2012).

32 E.g., for a compelling contemporary defence of the liberal view, see Jonathan Quong, *Liberalism without Perfection* (Oxford University Press 2011).

33 For an instructive overview, see Jeremy Waldron, ‘The Rule of Law’ *Stanford Encyclopedia of* Philosophy, (Fall 2023 Edition), Edward N. Zalta & Uri Nodelman (eds.), <https://plato.stanford.edu/archives/fall2023/entries/rule-of-law/>

34 The claim that necessity and proportionality analysis from self-defence ethics has important implications for speech governance is a central argument in Jeffrey W Howard, ‘Dangerous Speech’ (2019) 47 Philosophy & Public Affairs 208 and *Setting Fire to Reason: The Ethics of Free Speech *(Princeton University Press 2026).

35 Article 19(3), ICCPR.

36 Article 19(3) of the ICCPR is clear that the protection of other rights is a legitimate ground for interfering in freedom of expression, establishing that the right to freedom of expression can be subject to restrictions as long as these are provided by law and are necessary: (a) For respect of the rights or reputations of others; (b) For the protection of national security or of public order (*ordre public*), or of public health or morals. In the platform context, ‘provided by law’ is interpreted as provided under the rules of the platform.

37 Jeffrey W. Howard, ‘The Ethics of Social Media: Why Content Moderation Is a Moral Duty’ (2024) 11 Journal of Practical Ethics 33.

38 Douek suggests that because private platforms lack constitutional mandates, they simply could not claim the legitimacy that the proportionality test requires. But insofar as legitimacy refers to legitimate reasons for action to interfere with rights—rather than anything like representing a democratic will—it is not clear why the legitimacy question is inapplicable. On the limits of legitimacy in content moderation governance, more broadly, and for a criticism of the Oversight Board, in particular, see also Evelyn Douek, ‘The Meta Oversight Board and the Empty Promise of Legitimacy’ (2024) 37 Harvard Journal of Law & Technology.

39 David Kaye recognizes the possibility of social media companies’ deviating from the higher standards that govern state actions regarding freedom of expression, but emphasized that any deviations should be appropriately justified, and that such justification should draw upon IHRL. Kaye (n 28) para 48. Aswad, in turn, argues that even if SMCs are corporations, when adopting an IHRL framework they must abide by the same public interest assessments outlined in article 19(3) ICCPR; see Aswad (n 26).

40 See Ellie Rogers, ‘The Need for Greater Transparency in the Moderation of Borderline Terrorist and Violent Extremist Content’ (2025) 14 Internet Policy Review (arguing that transparency is especially critical for demotion measures precisely because they are harder for users to detect than overt removals).

41 See ‘The Santa Clara Principles on Transparency and Accountability in Content Moderation’ which states that platforms should have understandable and clear policies on ‘What types of content the company will take action against other than removal, such as algorithmic downranking’. Available at: https://santaclaraprinciples.org/

42 See, for example, Nicolas P Suzor and others, ‘What Do We Mean When We Talk About Transparency? Toward Meaningful Transparency in Commercial Content Moderation’ (2019) 13 International Journal of Communication 1528; Robert Gorwa and Timothy Garton Ash, ‘Democratic Transparency in the Platform Society’ in Nathaniel Persily and Joshua A Tucker (eds), *Social Media and Democracy: The State of the Field, Prospects for Reform* (1st edn, Cambridge University Press 2020); Paddy Leerssen, ‘The Soap Box as a Black Box: Regulating Transparency in Social Media Recommender Systems’ (2020) 11 European Journal of Law and Technology 51; Anna Lauren Hoffmann, ‘Where Fairness Fails: Data, Algorithms, and the Limits of Antidiscrimination Discourse’ (2019) 22 Information, Communication & Society 900.

43 C Thi Nguyen, ‘Transparency Is Surveillance’ (2022) 105 Philosophy and Phenomenological Research 331.

44 Suzor and others (n 42).

45 Bernhard Rieder and Jeanette Hofmann, ‘Towards Platform Observability’ (2020) 9 Internet Policy Review.

46 This is plausibly, in part, because Meta treats Hamas as a terrorist organization, such that content praising Hamas is prohibited; thus content that comes *close to praising Hamas (so-called borderline content) will get demoted.* We are grateful to an anonymous referee for helpful comments on this issue. See ‘Meta’s Broken Promises: Systemic Censorship of Palestine Content on Instagram and Facebook’ (Human Rights Watch 2023) <https://www.hrw.org/report/2023/12/21/metas-broken-promises/systemic-censorship-palestine-content-instagram-and>.

47 Gillespie notes platforms’ observation that demotion can be preferable to removal because it is less politically risky; but the controversies over shadowbanning show this is often not true. As discussed in Gillespie (n 1).

48 Similarly, Leerssen proposes a three-category framework for transparency, aimed at whom it's provided: user-facing disclaimers, government auditing, and data sharing. See Leerssen (n 5).

49 For a discussion of the ODS mechanism in the DSA and its limitations, see Beatriz Kira, ‘Regulatory Intermediaries in Content Moderation’ (2025) 14 Internet Policy Review.

50 See Amélie Heldt, ‘Borderline Speech: Caught in a Free Speech Limbo?’ [2020] Internet Policy Review.

51 See Gillespie (n 1); Leerssen (n 5).

52 See Virgílio Afonso Da Silva, ‘Standing in the Shadows of Balancing: Proportionality and the Necessity Test’ (2022) 20 International Journal of Constitutional Law 1738.

53 E.g., see Daniel Statman, ‘On the Success Condition for Legitimate Self‐Defense’ (2008) 118 Ethics 659.

54 On exposure being the mechanism by which speech harms, see JP Messina, ‘Public Calls for Censorship as Bad Speech’ (2022) 2 Journal of Free Speech Law 87.

55 What, exactly, counts as ‘necessary’ is complex, and philosophers have spilled a lot of ink exploring precisely that question, including the context of speech restrictions. See Howard (n 34).

56 For philosophical background, see Jonathan Quong, *The Morality of Defensive Force* (First edition, Oxford University Press 2020); Seth Lazar, ‘Necessity in Self‐Defense and War’ (2012) 40 Philosophy & Public Affairs 3. For a legal discussion, see Da Silva (n 52).

57 The Board has also invoked the necessity test in relation to its demonetisation policies for moderately (though not seriously) harmful speech, noting that ‘Meta should explore whether demonetisation is the least intrusive means of respecting the rights of vulnerable users’ (in Fruit Juice Diet case, 2023-019-FB-UA, 2023-020-FB-UA),

58 For an overview of relevant empirical evidence, see Pilar Aparicio-Martinez and others, ‘Social Media, Thin-Ideal, Body Dissatisfaction and Disordered Eating Attitudes: An Exploratory Analysis’ (2019) 16 International Journal of Environmental Research and Public Health 4177.

59 As noted in our discussion of legality, we lack a clear understanding from Meta of how it defines speech borderline to each community standard, so it is difficult to say for sure what counts as borderline hate speech.

60 It is a bit odd to think of racially insensitive jokes as borderline *hate speech*. We are tempted that platforms should ultimately dispense with the language of ‘borderline’ entirely, and simply specify the relevant categories of content under their listed demotion policies with appropriate labels.

61 Regina Rini, *The Ethics of Microaggression* (Routledge 2021).

62 Instructively, in TikTok’s ‘Eligibility Standards’ for its ‘For you Feed’, it notes: ‘Certain types of content may be fine if seen occasionally, but problematic if viewed in clusters’.

63 For prior discussion of these two forms of amplification (expansion vs. flooding), see Howard (n 37).

64 Indeed, research shows that people have robust psychological mechanisms for evaluating information, making them much less susceptible to persuasion than is often assumed. See, for example, Dan Sperber and others, ‘Epistemic Vigilance’ (2010) 25 Mind & Language 359; Hugo Mercier, ‘How Good Are We At Evaluating Communicated Information?’ (2021) 89 Royal Institute of Philosophy Supplement 257.

65 Even at a group level, research questions how much people's worldviews and beliefs are actually influenced by the information they are exposed to, suggesting that the link between misinformation and significant changes in a citizenry’s attitudes and behaviours is not as strong as is often assumed. See, for example, Dietram A Scheufele, Nicole M Krause and Isabelle Freiling, ‘Misinformed about the ‘Infodemic?’ Science’s Ongoing Struggle with Misinformation’. (2021) 10 Journal of Applied Research in Memory and Cognition 522.

66 Ciara Nugent, ‘YouTube Has Been ‘Actively Promoting’ Videos Spreading Climate Denialism, According to New Report’ *Time* (16 January 2020) <https://time.com/5765622/youtube-climate-change-denial/> accessed 10 July 2024; Kari Paul, ‘Climate Misinformation on Facebook ‘Increasing Substantially’, Study Says’ *The Guardian* (4 November 2021) <https://www.theguardian.com/technology/2021/nov/04/climate-misinformation-on-facebook-increasing-substantially-study-says> accessed 10 July 2024.

67 The Oversight Board suggests this logic: ‘In cases where users post information about COVID-19 treatments that contradicts the specific advice of health authorities and where a potential for physical harm is identified but is not imminent, the Board strongly recommends that Facebook adopt a range of less intrusive measures… *Down-ranking content, to prevent visibility in other users' News Feeds, might also be considered*’ (Oversight Board, case decision 2021-008-FB-FBR, emphasis added). Our analysis here supports a bolder position: demotion is in fact required for such content.

68 See Howard (n 37).

69 For example, some work in the philosophy of language compares the spread of hate speech to the spread of a virus—and in so doing makes reference to the idea that there are differential doses (or viral loads) leading to differential spread and/or infection rates; but unlike epidemiology, the relevant quantities remain deeply unclear due to a lack of relevant empirical evidence. See Mihaela Popa‐Wyatt, ‘Online Hate: Is Hate an Infectious Disease? Is Social Media a Promoter?’ (2023) 40 Journal of Applied Philosophy 788. For the kind of rigorous empirical study that would help bring precision to debates about the effects of speech, see David Yanagizawa-Drott, ‘Propaganda and Conflict: Evidence from the Rwandan Genocide *’ (2014) 129 The Quarterly Journal of Economics 1947.

70 We are grateful to a referee for raising this objection.

71 Miller (n 6).

72 We are grateful to Paddy Leerssen for pressing us on this point.

73 Evelyn Douek, ‘Governing Online Speech: From ‘Posts-As-Trumps’ to Proportionality and Probability’ (2021) 121 Columbia Law Review 759.

74 There are other reasons we have not considered. Gillespie also canvasses a range of reasons why platforms might be inclined to demote content. Gillespie (n 1).

75 See S Tsakyrakis, ‘Proportionality: An Assault on Human Rights?’ (2009) 7 International Journal of Constitutional Law 468 (criticising the use of proportionality in human rights adjudication based on the ‘impossibility of measuring incommensurable values’). For a reply to the incommensurability thesis, see Virgílio Afonso da Silva, ‘Comparing the Incommensurable: Constitutional Principles, Balancing and Rational Decision’ (2011) 31 Oxford Journal of Legal Studies 273 (arguing that ‘the presence of incommensurable values does not preclude the possibility of weighing or balancing rights; on the contrary, weighing or balancing rights is a procedure that increases comparability among principles’).

76 Howard (n 34).

77 In the philosophical literature, this is typically referred to as wide proportionality. Proportionality stricto sensu as used by lawyers arguably encompasses both.

78 Anthony McCosker and Ysabel Gerrard, ‘Hashtagging Depression on Instagram: Towards a More Inclusive Mental Health Research Methodology’ (2021) 23 New Media & Society 1899.

79 Thiago Dias Oliva, Dennys Marcelo Antonialli and Alessandra Gomes, ‘Fighting Hate Speech, Silencing Drag Queens? Artificial Intelligence in Content Moderation and Risks to LGBTQ Voices Online’ (2021) 25 Sexuality & Culture 700; Oliver L Haimson and others, ‘Disproportionate Removals and Differing Content Moderation Experiences for Conservative, Transgender, and Black Social Media Users: Marginalization and Moderation Gray Areas’ (2021) 5 Proceedings of the ACM on Human-Computer Interaction 1.

80 Carolina Are and Susanna Paasonen, ‘Sex in the Shadows of Celebrity’ (2021) 8 Porn Studies 411; Danielle Blunt and others, ‘Posting Into the Void’ (Hacking//Hustling 2020).

81 Carolina Are, ‘The Shadowban Cycle: An Autoethnography of Pole Dancing, Nudity and Censorship on Instagram’ (2022) 22 Feminist Media Studies 2002.

82 Jessica Sage Rauchberg, ‘#Shadowbanned Queer, Trans, and Disabled Creator Responses to Algorithmic Oppression on TikTok’ in Pain Paromita (ed), *LGBTQ Digital Cultures: A Global Perspective* (1st edn, Routledge 2022).

83 Brooke Erin Duffy and Colten Meisner, ‘Platform Governance at the Margins: Social Media Creators’ Experiences with Algorithmic (in)Visibility’ (2023) 45 Media, Culture & Society 285.

84 Evelyn Douek, ‘Content Moderation as Systems Thinking’ (2022) 136 Harvard Law Review 526.

85 Meta made headlines by amending its hate speech policies in January 2025 to allow various forms of hate speech–triggering a public outcry. Perhaps Meta had a point that banning these forms of hate speech went too far. But a more moderate step would have been to *demote* this speech. Mightn’t that have struck the balance? See Joel Kaplan, ‘More Speech and Fewer Mistakes’, Meta Newsroom, January 7, 2025, https://about.fb.com/news/2025/01/meta-more-speech-fewer-mistakes; for critical commentary, see Casey Newton, ‘Inside Meta’s dehumanizing new speech policies for trans people’, Platformer, January 9, 2025, https://www.platformer.news/meta-new-trans-guidelines-hate-speech/.

